# Supplementary figures and images for: The abundance of large, piscivorous Ferox Trout (Salmo trutta) in Loch Rannoch, Scotland
Source: PeerJ. 2016 Nov 1;4:e2646. doi: 10.7717/peerj.2646 (PMC5101599; doi:10.7717/peerj.2646)

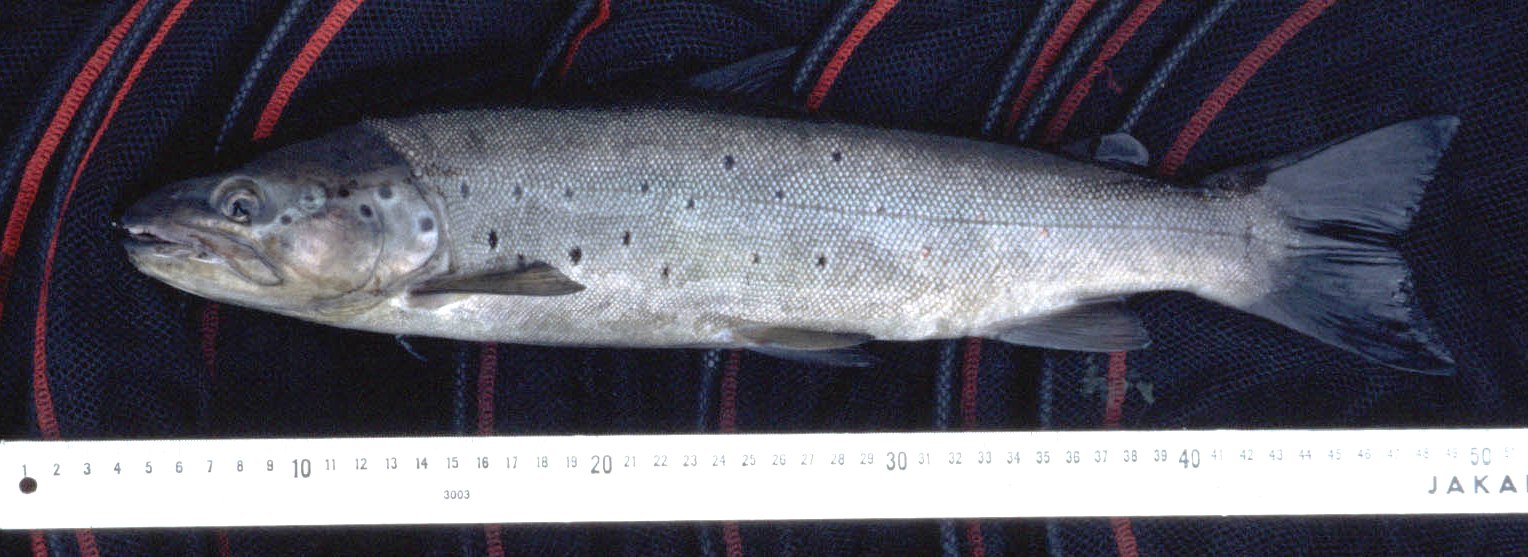

Supplement: Figure S1 [file peerj-04-2646-s001.jpg]

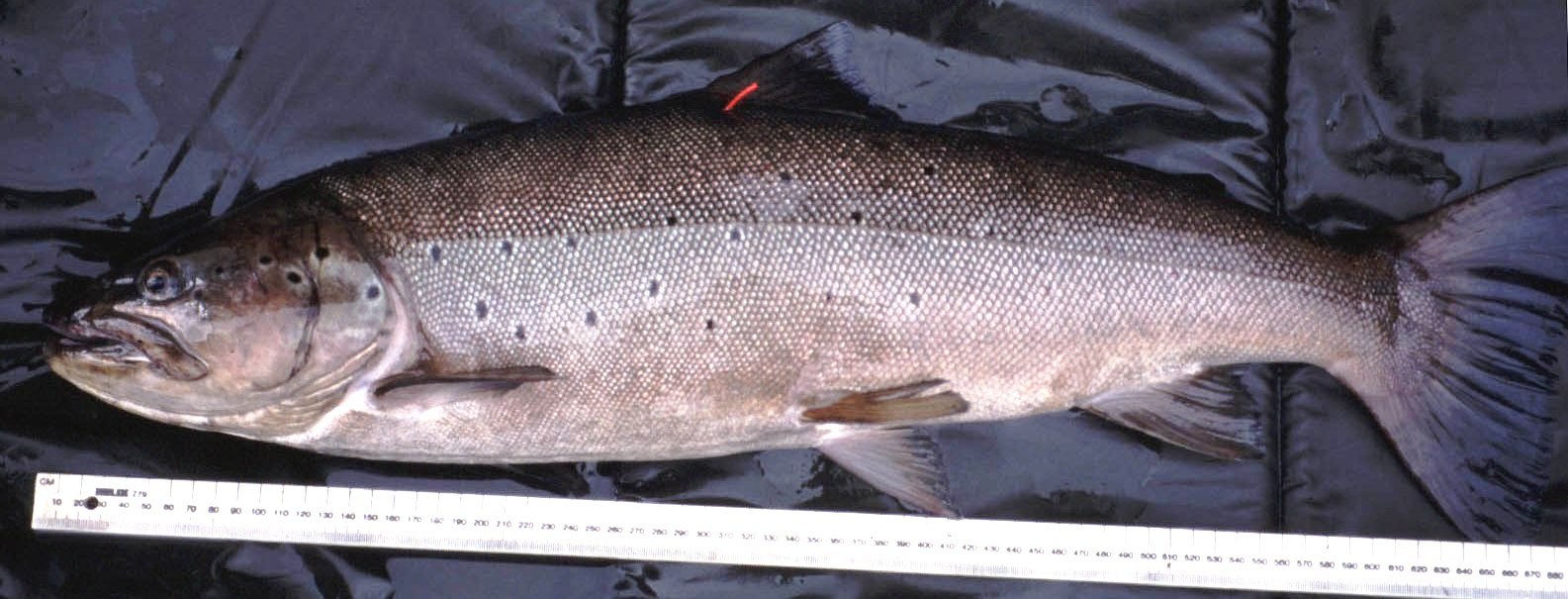

Supplement: Figure S2 [file peerj-04-2646-s002.jpg]

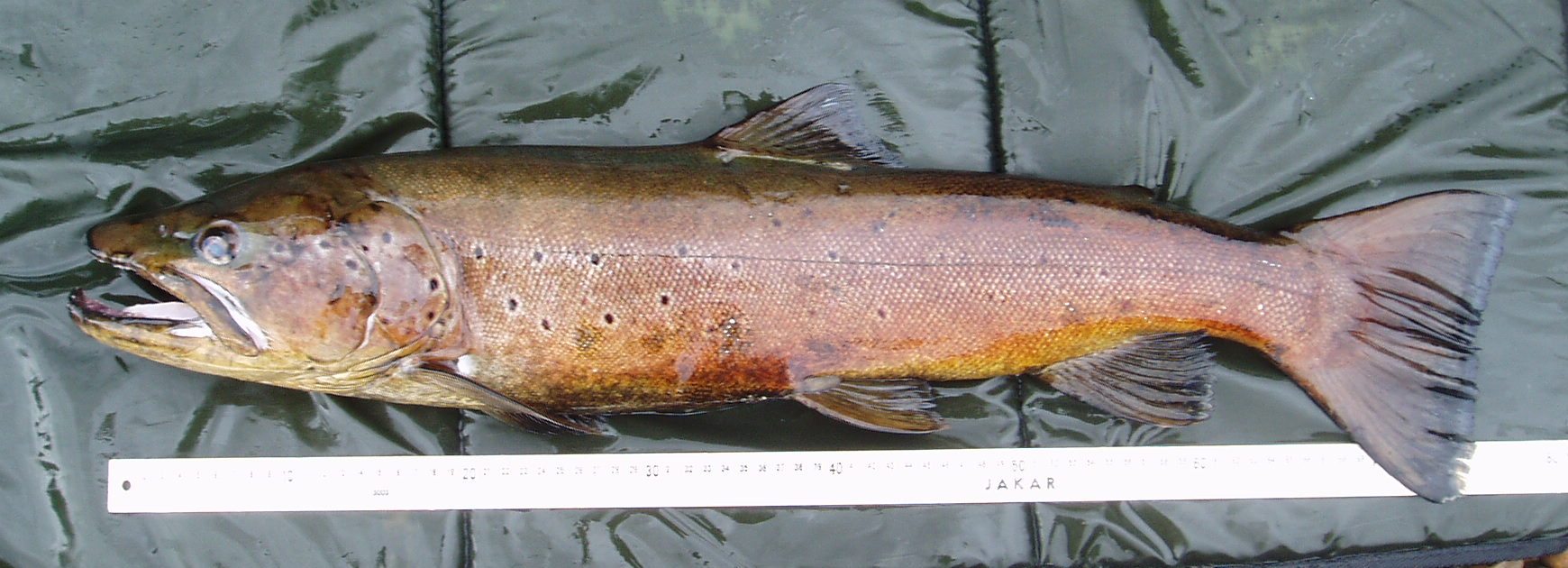

Supplement: Figure S3 [file peerj-04-2646-s003.jpg]
