# Supplementary material for: The abundance of large, piscivorous Ferox Trout (Salmo trutta) in Loch Rannoch, Scotland
Source: PeerJ. 2016 Nov 1;4:e2646. doi: 10.7717/peerj.2646 (PMC5101599; doi:10.7717/peerj.2646)
Supplement: Article S1 [file peerj-04-2646-s004.pdf]

To replicate the analyses

1. Install R Version (>= 3.2.2) from https://cran.r-project.org.
2. Install JAGS (>= 3.4.0) from http://sourceforge.net/projects/mcmc-jags/files/ to the default location.
3. If using Windows install Rtools (>= 3.2.2) from https://cran.r-project.org/bin/windows/Rtools/ choosing the default options.
4. Cut and paste the following code into the R console to quickly generate non-convergent results.

```
install.packages("devtools")
library(devtools)

install_github("poissonconsulting/tulip@v0.0.13")
install_github("poissonconsulting/datalist@v0.5.1")
install_github("poissonconsulting/juggler@v0.1.5")
install_github("poissonconsulting/jaggernaut@v2.3.3")

install_github("poissonconsulting/ranmrdata")
install_github("poissonconsulting/ranmr")

library(ranmr)
replicate_results()
```

5. Open the `results` folder in the working directory to view the plots which are saved as png files.
6. For more information type `?replicate_results`.
